# Effect of triploidy on liver gene expression in coho salmon (*Oncorhynchus kisutch*) under different metabolic states

**DOI:** 10.1186/s12864-019-5655-8

**Published:** 2019-05-03

**Authors:** Kris A. Christensen, Dionne Sakhrani, Eric B. Rondeau, Jeffery Richards, Ben F. Koop, Robert H. Devlin

**Affiliations:** 1 Fisheries and Oceans Canada, West Vancouver, BC Canada; 2 Department of Zoology, University of British Columbia, Vancouver, BC Canada; 3 Department of Biology, University of Victoria, Victoria, BC Canada

**Keywords:** Ploidy, Triploid, Gene dosage, Transgenic, RNA-seq, Salmonid, Growth hormone

## Abstract

**Background:**

Triploid coho salmon are excellent models for studying gene dosage and the effects of increased cell volume on gene expression. Triploids have an additional haploid genome in each cell and have fewer but larger cells than diploid coho salmon to accommodate the increased genome size. Studying gene expression in triploid coho salmon provides insight into how gene expression may have been affected after the salmonid-specific genome duplication which occurred some 90 MYA. Triploid coho salmon are sterile and consequently can live longer and grow larger than diploid congeners in many semelparous species (spawning only once) because they never reach maturity and post-spawning mortality is averted. Triploid fishes are also of interest to the commercial sector (larger fish are more valuable) and to fisheries management since sterile fish can potentially minimize negative impacts of escaped fish in the wild.

**Results:**

The vast majority of genes in liver tissue had similar expression levels between diploid and triploid coho salmon, indicating that the same amount of mRNA transcripts were being produced per gene copy (positive gene dosage effects) within a larger volume cell. Several genes related to nutrition and compensatory growth were differentially expressed between diploid and triploid salmon, indicating that some loci are sensitive to cell size and/or DNA content per cell. To examine how robust expression between ploidies is under different conditions, a genetic/metabolic modifier in the form of different doses of a growth hormone transgene was used to assess gene expression under conditions that the genome has not naturally experienced or adapted to. While many (up to 1400) genes were differentially expressed between non-transgenic and transgenic fish, relatively few genes were differentially expressed between diploids and triploids with similar doses of the transgene. These observations indicate that the small effect of ploidy on gene expression is robust to large changes in physiological state.

**Conclusions:**

These findings are of interest from a gene regulatory perspective, but also valuable for understanding phenotypic effects in triploids, transgenics, and triploid transgenics that could affect their utility in culture conditions and their fitness and potential consequences of release into nature.

**Electronic supplementary material:**

The online version of this article (10.1186/s12864-019-5655-8) contains supplementary material, which is available to authorized users.

## Background

Polyploidy (multiple genome copies within a single cell) is a common phenomenon found throughout all domains of life and likely predates multicellular organisms (e.g. through endopolyploidy, reviewed in [1]). Polyploid organisms are common in the plant kingdom [2], Archaea and Bacteria domains [3–5], and in amoebae species [6]. To better understand polyploidy, it is useful to consider the transition from haploid to diploid phases in sexually reproducing organisms, as this is considered a special form of genome duplication (autopolyploidy) that can lead to meiotic sexual reproduction.

In a broad sense, all organisms with meiotic sexual reproduction change between haploid (a single copy of the genome per cell, 1 N) and diploid (two genome copies per cell, 2 N) states/phases. The dominant ploidy can be haploid (e.g. some insects), diploid (e.g. humans), or alternate between ploidy states (e.g. alga) (reviewed in [7–10]). Some species, which alternate between dominant ploidies, can have very similar phenotypes (e.g. alga) [8]. It is thought that there are evolutionary benefits of both ploidies. Depending on context, haploids can adapt faster than diploids, while diploids will accumulate more variation (as there are more targets for mutation and opportunity for rearrangement of alleles), which generates increased genetic potential [8, 10]. The transition from a haploid phase to a diploid phase is often characterized by an increased tolerance of negative mutations and cell size [8, 10].

Similarly, diploid tissues have characteristics that are adaptive in some environments and polyploid tissues are adaptive in others. Several human and mouse tissues commonly have a contingent of polyploid cells (e.g. heart, placenta, and brain). In the liver, polyploid cells are generated through several mechanisms and may be an adaptive strategy to cope with toxic compounds (reviewed in [11]). Tissue specific polyploidy, commonly referred to as endopolyploidy, may be beneficial in a tissue as it generates much larger cells (i.e. hypertrophy) [1, 12], and polyploid tissues may be more resistant to stress [13]. Hypertrophy, in turn, may have repercussions on mechanical aspects of a cell, as in the case of cells (e.g. megakaryocytes) migrating between microporous barriers (reviewed in [14]). It may also have detrimental implications for spindle formation, which is important for chromosomal segregation during cell division [14].

Gene expression studies between tissues of diploid and polyploid organisms report similar results to cell-based expression studies (from diploid and polyploid tissues of the same organism). In general, transcript number proportionally increases with ploidy, also known as positive gene dosage effects, and remains constant relative to each chromosome with a few exceptions [12, 15–21]. This is in contrast to dosage compensation when only part of the genome is duplicated and gene expression patterns are drastically altered [21–24]. Early studies using Drosophila found that gene product levels were similar between diploids and triploids, indicating that the relationship between gene dosage, cell size, and gene product remain approximately in balance [25, 26]. More recently, gene expression studies between diploid and polyploid plants revealed relatively few genes that were differentially expressed between ploidies [27–29].

Whole-organism polyploidy has been a significant force in vertebrate evolution even though there are few extant vertebrate polyploids (e.g. sturgeon, salmon, carp, minnows, mollies, frogs, toads, salamanders, newts, etc. reviewed in [7]). Vertebrates share ancestral genome duplications that likely influenced the trajectory of their evolution. Two whole-genome duplications, commonly referred to as the 1R and 2R genome duplications, occurred early in vertebrate evolution, and some remnants of these duplications can still be found in their/our genomes [30–32]. This early vertebrate polyploidy is thought to have influenced body plan and organ complexity.

Salmonids possess an additional two genome duplications. One is commonly referred to as the 3R genome duplication, and is shared with all teleosts [33]. The other is referred to as the 4R genome duplication and is specific to the Salimoniformes lineage [33]. Many of the pairs of chromosomes duplicated in the 3R genome duplication can still be identified through synteny or double conserved synteny [32, 34]. Some of the duplicated regions from the most recent duplication (4R) are nearly identical in sequence (autopolyploidy) [34–36] due to residual tetrasomic inheritance [37, 38]. The majority of genes have been retained in duplicate [36], and as such, some have specialized, gained new functionality, or have remained primarily unchanged [36]. With salmonids’ rich history of thriving following genome duplication, they provide an interesting model to examine gene regulatory mechanisms that have allowed them to survive, reproduce successfully, and to diversify.

The production of triploid salmonids began in the early 1980’s, when it was discovered that a large fraction of artificially produced triploid salmon and trout were able to survive without any major differences in phenotype to diploids [39–42]. Triploid juvenile coho salmon have similar growth rates as diploids (among other traits [42]) and respond similarly after growth hormone (GH) treatment, however, GH-triploids had significantly higher moisture and lower lipid content [43]. In a similar study, a GH transgene (integrated into the genome) was used in a dosage series (zero, one, or two doses in diploids, and zero, one, two, or three doses in triploids) to measure growth of diploid and triploid coho salmon [44]. Non-transgenic diploids and triploids had similar growth, whereas transgenic triploids were smaller than transgenic diploids for all comparable dosages of the transgene [44]. These studies suggest that triploid coho salmon may be less adept at adjusting to a stressor than diploids, at least in the form of increased growth rate. When differentially expressed genes were examined between diploid and triploid liver tissue of bighead catfish [45] five of the top 20 upregulated genes were found to be involved in responding to stressors. Compensatory mechanisms may be adequate for normal growth of triploids, but fail when additional strains are placed upon triploid fish as suggested from an immune challenge experiment with diploid and triploid Chinook salmon [46].

Liver tissue was chosen in this study as it regulates energy metabolism and would likely be ideal for studying why growth differences exist between diploid and triploid coho under stressful conditions. Coho salmon liver tissue is composed of cells of a single ploidy, either diploid in diploid coho salmon or triploid in triploid coho salmon [40], unlike mosaic human and mice liver tissues that are composed of diploid, tetraploid, and octoploid cells (discussed above). Liver tissue from coho salmon possessing different dosages (zero to three) of a growth hormone transgene were utilized for RNA extraction and transcriptome analysis [44]. By understanding the physiological and gene expression consequences of triploidy (alone or in conjunction with other technologies such as transgenesis), evolutionary consequences of polyploidy in salmon can be examined and add to our insight into what happens after whole genome duplication. In addition, risks and benefits associated with these genetic methods can be better assessed within fisheries and environmental management goals, as well as in commercial contexts for both production and animal welfare objectives.

## Results

### Alignment statistics

The average number of sequenced reads in this study was nearly 31 million per individual, and ~ 23 million of those sequences uniquely aligned to the coho genome (Additional file 1: Table S1, Additional file 2). Approximately 85% of all sequences (both paired and non-paired) aligned to the genome in this study (Additional file 1: Table S1, Additional file 2) and around 76% aligned to the genome as a pair. These values are typical of RNA-seq experiments, arising from elimination of reads that map equally well to different regions (reviewed in [47]).

### Global gene expression among groups

**Fig. 1:**
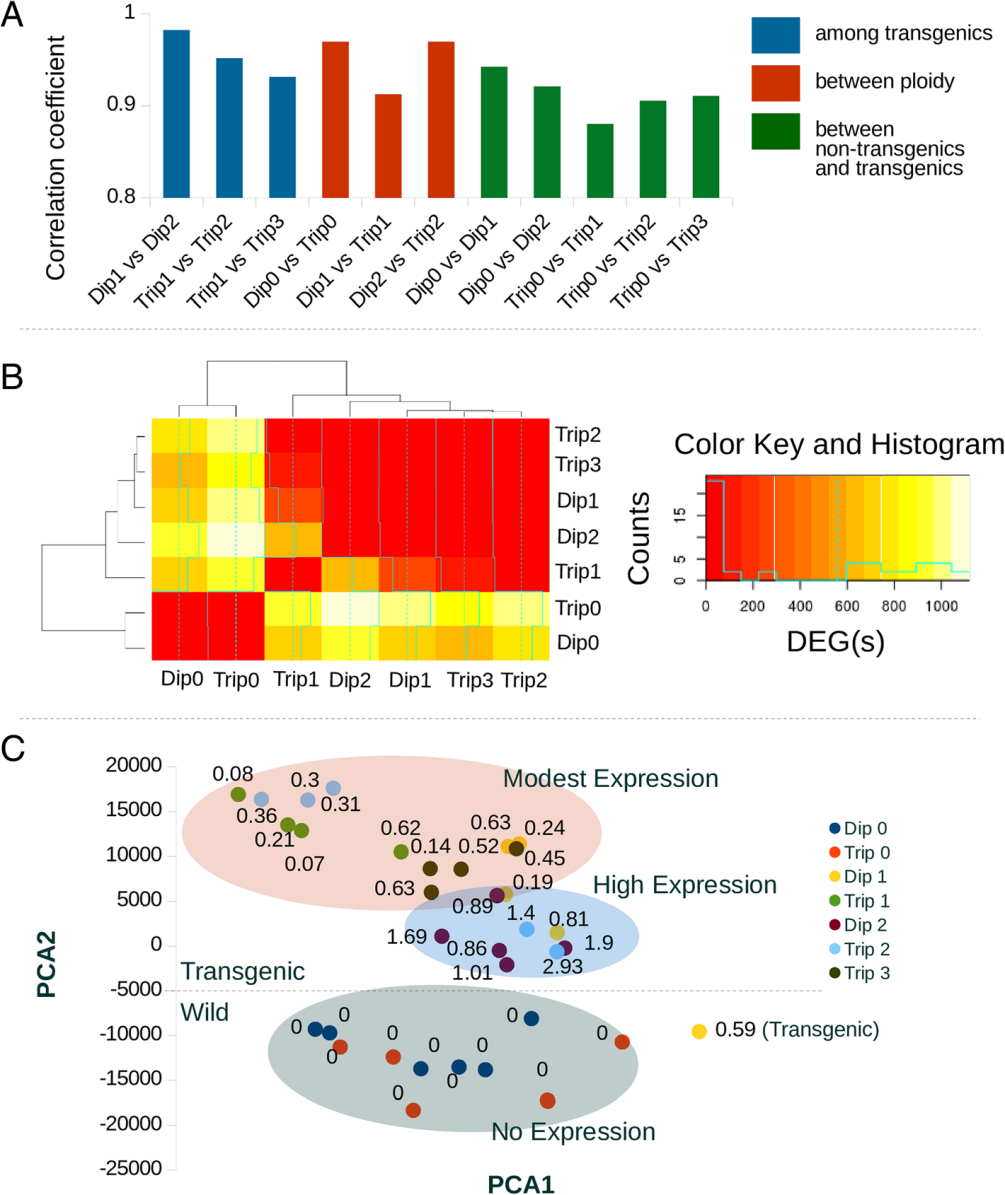
Gene Expression Correlation, DEG Counts, and PCA Among Groups. **a** The gene expression (of all genes) correlation coefficient, sorted from high to low, among all groups of coho salmon with different genotypes. Groups with “Dip” in their names are diploid, while those with the “Trip” in their names are triploids. The different numbers in each name represents the number of transgenic growth hormone genes in the genomes of that group. **b** A heatmap of the number of shared DEG(s) between different groups produced in R [85] using the heatmap.2 function in [86]. **c** A plot of the first two largest principal components of a global gene expression principal component analysis. All individual coho salmon are plotted with the colour representing the group to which they belong to. Growth hormone gene expression (FPKM) is overlaid to highlight patterns of groupings based on growth hormone rather than genetic background. The dotted line indicates where most individuals are separated as either wild or transgenic, only one transgenic individual (a Dip1) is associated with wild fish (indicated on the figure)

To understand large-scale relationships of gene expression among the different groups, correlation coefficients of global gene expression values were calculated between groups, the numbers of differentially expressed genes (DEGs) identified among groups were counted, and a principal component analysis was performed (Fig. 1). All pairwise comparisons between groups had high global gene expression correlation coefficients with a slight trend of lower correlation (Fig. 1a) and a large number of DEGs among non-transgenic and transgenic groups (Fig. 1b, Table 1). Individuals from groups that had similar GH transgene doses tended to cluster in the principal component analysis plot (Fig. 1c). When the expression level of the growth hormone gene was overlaid onto the principal component plot, three clusters were found to explain the placement of most individuals (Fig. 1c). No genes were significantly differentially expressed between Trip2 and Trip3 in the liver (Fig. 1a, Table 1).

**Table 1:** DEGs Counts and Direction of Expression Differences Between Groups

	Dip0	Dip1	Dip2	Trip0	Trip1	Trip2	Trip3
Dip0		+ 200/− 536	+ 356/− 594	+ 6/− 10	+ 333/− 390	+ 306/− 467	+ 197/− 409
Dip1	736		+ 27/−4	+ 737/− 277	+ 249/− 25	+ 1/− 1	+ 2/− 3
Dip2	950	31		+ 734/− 383	+ 381/− 234	+ 0/− 5	+ 0/− 1
Trip0	16	1014	1117		+ 376/− 530	+ 375/− 598	+ 298/− 553
Trip1	723	274	615	906		+ 16/−26	+ 39/− 63
Trip2	773	2	5	973	42		+ 0/−0
Trip3	606	5	1	851	102	0	

In cells below the diagonal (with single numbers), the number of DEGs are shown between groups. In cells above the diagonal, the number of upregulated (+) and downregulated (−) DEGs relative to the group in the matching row are shown. Groups with “Dip” in their names are diploid, while those with the “Trip” in their names represent triploids. The different numbers in each name represents the number of transgenic growth hormone gene doses in the diploid or triploid genome of that group

### Comparison between ploidies

**Fig. 2:**
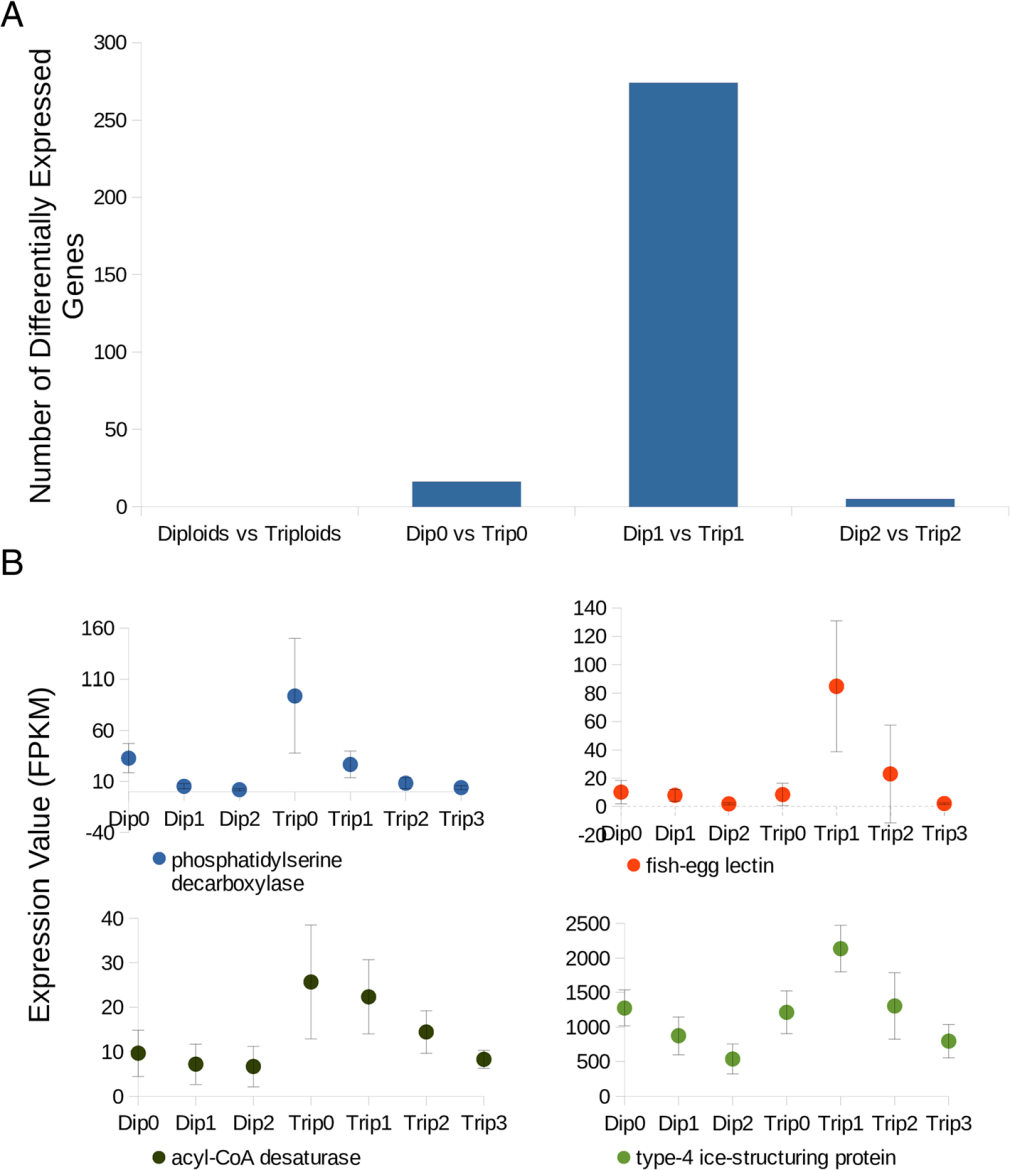
Gene Expression Patterns Between Groups with Different Ploidy. **a** Bar graph showing the number of differentially expressed genes between coho salmon groups with different ploidies. The first category (i.e. diploids vs triploids) is a comparison between all diploids and triploids (including triploids with three copies of the transgene). The other categories compare diploid and triploids pairs with the same copy number of the transgene. **b** Graphs of the differentially expressed genes that are shared between the different comparisons from A, showing the average gene expression level for the different groups (with error bars representing the standard deviation)

Overall, there were no genes that were consistently differentially expressed between all diploid and triploid fish in this study (left-most bar in Fig. 2a). Between Dip0 and Trip0 fish, 16 DEGs were identified (Table 1, Additional file 1: Table S2, Additional file 2), with only two of these 16 overlapping (acyl-CoA desaturase also known as stearoyl-CoA desaturase and phosphatidylserine decarboxylase) with the 274 DEGs found between the Dip1 and Trip1 groups (Fig. 2b, Additional file 2), and none overlapped with the five DEGs found between the Dip2 and Trip2 groups. Most of the 274 DEGs found between the Dip1 and Trip1 groups were upregulated in diploids (Table 1, Additional file 2). Fish-egg lectin and type-4 ice-structuring protein were both differentially expressed in the Dip1 vs. Trip1 and the Dip2 vs. Trip2 comparisons.

### Growth hormone transgene expression

**Fig. 3:**
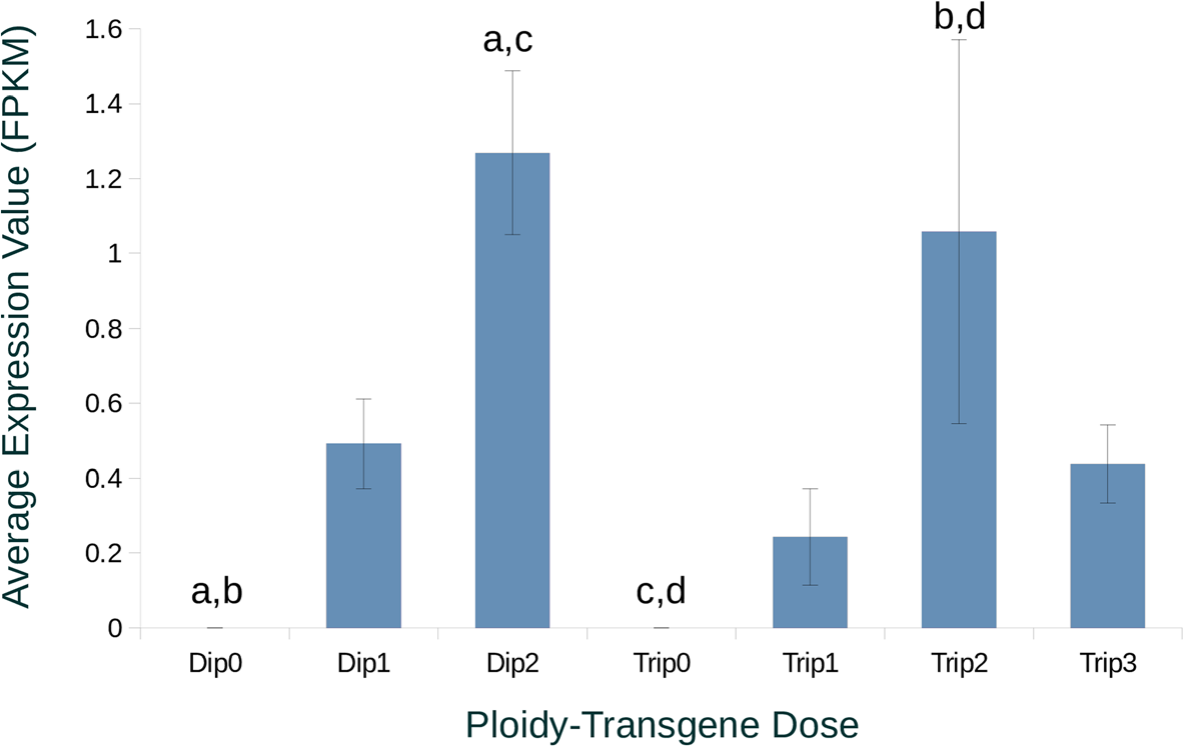
Transgenic Growth Hormone Gene Expression in Different Groups of Coho Salmon. The average copy number (FPKM ± SE) of growth hormone (NCBI Gene ID: 109893213) of different coho salmon with different genetic backgrounds. Groups with “Dip” in their names are diploid, while those with the “Trip” in their names represent triploids. The different numbers in each name represents the number of transgenic growth hormone gene doses in the diploid or triploid genome of that group. Matching letters represent significant differences (before false discovery rate controlling procedures) in FPKM between groups. There were no significantly different groups after false discovery procedures

The Dip0 and Trip0 groups had undetectable levels of hepatic growth hormone gene expression (Fig. 3), which is consistent with them lacking the growth hormone transgene. Expression of the growth hormone 2 gene (LOC109898300) was also assessed (Additional file 2), but had extremely low expression for all categories as expected for non-pituitary tissue (the few reads that were detected were likely due to erroneous mappings from the transgenic growth hormone to growth hormone 2 because of high sequence similarity between the coho salmon genome GH2 gene and the sockeye salmon GH2 present in the transgene). Dip1 and Trip1 appeared to have an intermediate GH expression level between groups without the transgene and those with two copies, however, only the groups without the transgene and those with two copies had significantly different expression (fragments per kilobase of transcript per million mapped reads or FPKM) (Fig. 3). Transgenic triploid coho salmon had a trend of lower GH gene expression (FPKM) for comparable doses of GH without being significantly different.

### Effects of the growth hormone transgene

**Fig. 4:**
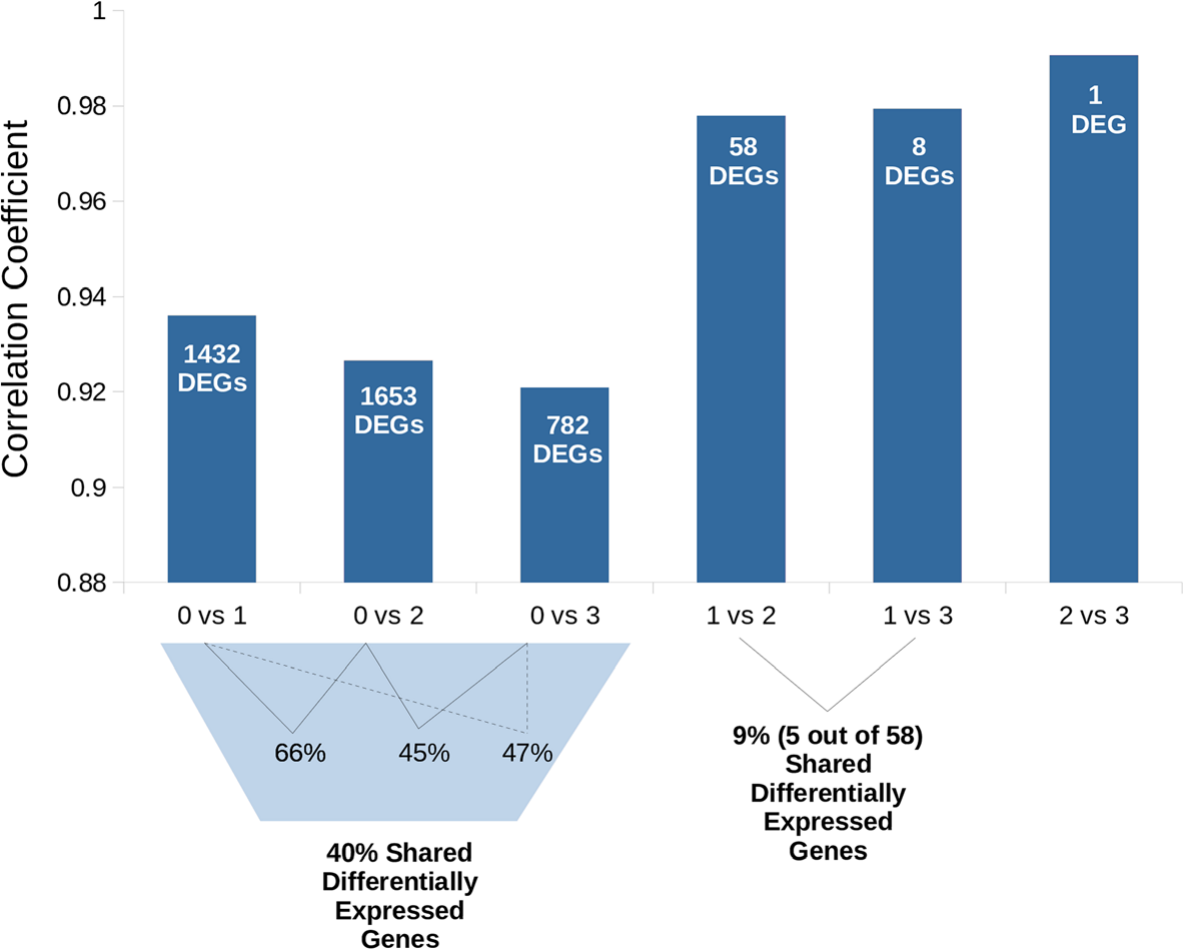
Gene Expression Patterns Among Groups with Different Copies of Transgenic Growth Hormone Gene. A comparison of correlation coefficients between different coho salmon groups based on the copy number of the growth hormone gene. The numbers on the x-axis represent both diploid and triploid groups that have either 0, 1, 2, or 3 copies of the transgene (3 copies only possible in the triploid group). The white numbers within bars represent the number of significantly differentially expressed genes (after false discovery rate controlling procedures) between indicated groups. The percentages shown below the graph, depict the number of overlapping differentially expressed genes between comparisons

Non-transgenic coho salmon groups had lower global gene expression correlation coefficients with transgenic groups. Further, non-transgenic/transgenic comparisons revealed many more (> 10 fold) DEGs than any of the comparisons among transgenic groups with different transgene doses (Fig. 4). A large number (40%) of the DEGs identified among non-transgenic and transgenic groups overlapped (Fig. 4). There were 656 enriched gene ontology (GO) categories found among the DEGs between Dip0 and Dip1, and 842 between Trip0 and Trip1 (Additional file 3: Figure S1, Additional file 4: Figure S2, Additional file 2 for DEG numbers in GO categories). Many of the genes differentially expressed between non-transgenic and transgenic coho salmon are related to metabolism, oxidation-reduction process, oxygen transport, response to stress, and mitochondrion organization (Additional file 3: Figure S1, Additional file 4: Figure S2, Additional file 1: Tables S3-S4, Additional file 5: Figure S3, Additional file 6: Figure S4, Additional file 2). The majority of genes in these categories were upregulated in transgenic fish and related to metabolism (Additional file 1: Tables S3-S4). In contrast, only three GO terms were enriched in the comparison between transgenic fish with one copy versus two copies of the growth hormone transgene (GO:0055114: oxidation-reduction process, GO:0043390: aflatoxin B1 metabolic process, GO:0042448: progesterone metabolic process). SPARC-like protein 1 (or Hevin) was the only DEG found between transgenic fish with two copies of the GH transgene and those with three (elevated in fish with three copies).

Of the 274 DEGs between Dip1 and Trip1 (Table 1, Additional file 7: Figure S5, Additional file 2), 145 were unique to this comparison and not found in the comparison between Dip0 and Dip1 or between Trip0 and Trip1 (Additional file 2). This unique set represents genes responsible for the differences seen between Dip1 and Trip1 growth [42], while removing the genes that are commonly differentially regulated during GH transgenesis and the production of triploid coho salmon. The majority of these genes were found to code for ribosomal proteins (115 genes, ~ 80%), and the DEG with the lowest *p*-value was glucose-6-phosphatase-like (Additional file 1: Table S5). In this comparison, 277 enriched GO categories were identified (Additional file 7: Figure S5, Additional file 8: Figure S6, Additional file 1: Tables S3-S4, Additional file 2). Translation, ribosome biogenesis, and metabolism were the three most significant categories in the tree map (Additional file 7: Figure S5). Translation and ribosome biogenesis related genes tended to be upregulated in diploid transgenic coho salmon (Additional file 1: Table S4).

## Discussion

In this study, gene expression in liver tissue from diploid and triploid coho salmon was used to assess the degree of change that occurs as a result of ploidy manipulation. These effects were also analyzed in fish possessing different doses of a GH transgenic locus to assess whether ploidy effects were sensitive to altered genetic and physiological conditions. Hepatic gene expression was the same between all diploid and triploid individuals when analyzed together, but differed from a few genes to a few hundred genes when examining pairwise comparisons of diploid and triploid fish matched by GH transgene dose. Much greater differences were observed among comparisons of non-transgenic and GH transgenic coho salmon, regardless of ploidy. Few DEGs were found among comparisons of GH transgenic fish with various doses of the GH transgene.

The few differences seen between diploid and triploid fish is consistent with proteomic analysis of liver tissue between diploid and triploid Atlantic salmon from alevin to parr stages [48], and positive genome dosage effects (see Background) balanced with fewer, larger cells per unit of liver tissue found in triploids [42]. Indeed, there were no GO categories that were enriched between the Dip0 and Trip0 comparison indicating genome dosage effects are well balanced by changes in cell volume and number that equalizes gene regulatory mechanisms between ploidies for the majority of the genome.

The few differences in gene expression that were detected between diploid and triploid coho were similar to findings in bighead carp [45], where two of the top 20 upregulated genes found in triploid bighead catfish (Eukaryotic translation initiation factor 4, and ATP-binding cassette subfamily F member 2-like) were also upregulated in the coho salmon triploids (Additional file 1: Table S2). Kerr [49] found that ATP-binding cassette subfamily F members may have functions in translation initiation and elongation. If that is the case, three of the 16 DEGs between Dip0 and Trip0 were involved in translation initiation, and all were upregulated in triploids. Likewise, nucleolar protein 58 (NOP58) is involved in ribosome biogenesis [50], as is nucleophosmin among other roles [51, 52]. Both genes are upregulated in the coho salmon triploids. In fasted and refed trout, a similar pattern of translation initiation and ribosome biogenesis was seen [53] with many fewer DEGs reported here.

Stearoyl-CoA desaturase (also known as acyl-CoA desaturase) was found to be upregulated in triploid fish and may indicate nutritional deficiency in triploid fish. In mice, upregulation of stearoyl-CoA desaturase was associated with the resumption of feeding after fasting (reviewed in [54]). In a study on the effects of a plant-based diet on the transcriptome profile of liver tissue from European sea bass, stearoyl-CoA desaturase showed a 1.4-fold increase in fish that were fed a plant-based diet (which was associated with lower growth) relative to fish meal/oil supplemented feed [55]. Geay [55] also found that expression of the phosphatidylserine decarboxylase proenzyme gene, another DEG between Dip0 and Trip0, in the fish fed the plant-based diet was 2.8 times higher than the expression in fish fed a fish meal/oil supplemented feed. Upregulation of both these genes in sea bass was associated with a plant-based diet and lower growth, suggesting that their upregulation might be an effect of malnutrition. Taken together, the genes that were differentially expressed between Dip0 and Trip0 illuminate a snapshot of a potential nutritional deficiency in triploid fish. This information may be useful in future studies researching the development of fish feeds for triploids and points to additional nutritional requirements associated with triploidy. These results also suggest that in stressful or nutritionally deprived environments (that might be expected periodically in some high-density fish culture or in the wild), triploid fish might be at a disadvantage. If this is the case, then it has important implications for their suitability to aquaculture and for the level of risk they may pose to the natural environment.

This data provides context to the long history of polyploidy in salmonids. The coho salmon in this and previous studies (see Background) all seemed able to cope well with an extra genome copy since their growth and hepatic gene expression varied little from diploids. Positive gene dosage effects balanced by fewer, larger cells may explain why the fish in this study were little impacted by ploidy. These data provide evidence that mechanisms exist at the gene expression, cell size, and organismal (cell number per unit amount of tissue) levels to allow compensation for different ploidies, and by extension, how major evolutionary events such as the 4R genome duplication was able to persist in the ancestors to salmonids. Initially, there would likely be little difference in terms of gene expression, and the larger cells in tetraploids would likely have roughly the same concentrations of transcription factors and hence mRNAs (see Background). This hypothesis may explain how tetraploidy initially persisted, but as genes were lost or gained new functions though time (i.e. diploidization), there would likely be changes in gene expression patterns similar to autosomal dosage compensation seen in response to aneuploidy [15, 21, 23, 24].

In contrast to the minimal effects arising from changes in ploidy, GH transgenesis generated much greater changes in growth and gene expression compared to non-transgenic fish. This result is consistent with other studies where the growth hormone transgene triggers a substantially different expression profile in the liver of salmonids [56–58]. From a previous but related study examining ploidy effects on endocrine function in the GH/IGF-I axis [44] (Additional file 9: Figure S7, reproduced with permission), it was seen that at 201 days post first feeding, non-transgenic diploid and triploid coho have similar weights under common garden rearing conditions. With the addition of GH transgenesis, triploid salmon growth was lower compared to diploid coho salmon for all comparable GH transgene doses, but still increased with dose (Additional file 9: Figure S7). Interestingly, triploid coho salmon with three copies of the GH transgene weighed the same as triploids with two copies of the transgene. A similar trend was seen in the expression of GH in triploids with three copies of the transgene (Additional file 9: Figure S7). These results show positive gene dosage effects on transcript number from the transgene until there are three copies of the transgene.

The reduction of GH gene expression (FPKM) in triploids with three copies of the GH transgene, suggests dosage-influenced transcriptional regulation. Dosage-influenced gene silencing (reduction of expression, not necessarily complete inactivation) has been seen in other transgenic organisms [59, 60], where an increased number of transgenes causes silencing of the transgene. A possible mechanism for transgene silencing is RNA-mediated inhibition triggered by a threshold concentration [60]. The decrease in GH gene expression (FPKM) observed in the present study and equivalent weights between Trip2 and Trip3 found in a previous study support dosage-influenced gene silencing in triploid coho with three copies of the transgene.

Autosomal dosage compensation mechanisms in response to aneuploidy in Drosophila do not appear to function by silencing the expression of alleles of a gene [61, 62], but rather appear to be transcriptionally regulated and operate at the individual gene level (including transgene inserts) rather than on chromosome segments [21, 24, 61, 62]. It has been proposed that regulatory loci acting in a negative fashion operate to suppress the expression of genes in a trans fashion [21, 23, 63]. In a ploidy series where gene balance across the genome is maintained, such regulation is expected to have little effect as seen in triploid/diploid comparisons, but as mentioned above, when piecemeal genome rediploidization ensues, adjustments in the expression of regulatory loci likely play significant roles in allowing modulation of gene product levels to maintain viability of the organisms.

The enrichment of so many GO categories related to metabolism in the present study is consistent with the known function of GH. The results and GO categories were quite similar between comparisons of transgenic and non-transgenic groups both in diploid and triploid fish, suggesting a common mechanism in both ploidies. The GO term categories offer non-specific indicators of growth hormone transgenesis and provide a baseline for comparisons to other transgenic fish strains (e.g. identifying escaped transgenic fish or those exposed to exogenous GH). These data also indicate that, relative to ploidy differences, the effects of the growth hormone transgene are very potent at modifying physiological pathways and phenotype relative to wild fish (e.g. the liver gene expression profile is extensively altered, > 1400 DEGs), with even a single transgene copy.

To better understand why transgenic triploids did not grow as well as transgenic diploids, DEGs were identified that were found only in the Dip1 vs. Trip1 comparison and not in the Dip0 vs. Dip1 or Trip0 vs. Trip1 comparisons. Removing the common DEGs, allowed us to examine expression differences that are the result from the interplay between the transgene and ploidy rather than an effect of either alone. Glucose-6-phosphatase had the lowest *p*-value in this unique set. The glucose-6-phosphatase protein is known to provide glucose under starvation conditions by elevating glucose-6-phosphatase mRNA in the liver (reviewed in [64]). Further, GH transgenic coho salmon have been shown to have modifications to glucose metabolism pathways and have a greater capacity to utilize dietary carbohydrate [65–67]. The dysregulation of glucose metabolism, the reduction of ribosomal producing genes, and the reduction of translation related genes again implicate a need for enhanced production of energy (i.e. an induced nutritional deficiency) as a major difference between Dip1 and Trip1 growth. In diploids, there is a significant increase in glucose-6-phosphatase after transgenesis, but not in triploids (Additional file 2), suggesting that diploid coho salmon are compensating for the increased metabolic demands of increased GH in a different mechanism than triploids.

Transgenic GH gene dosage between diploid and triploid coho salmon likely influences hepatic gene expression and growth between Dip1 and Trip1 as well. There is one GH transgene for the two and three haploid genomes in the Dip1 (1:2) and Trip1 (1:3) individuals, respectively. Assuming positive gene dosage effects (which is well supported in the present study and previously), there would be fewer transgenic GH transcripts per cell volume in the Trip1 group than the Dip1 group. The difference in GH expression (FPKM), though not significant in the present study, was lower in the Trip1 group; it is unlikely that the fractional difference of 1/6th dose would reach significance with the current sample size or with multiple testing correction. The hypothetical difference in GH transgene concentration between Dip1 and Trip1 could explain the many more DEGs found in the Dip1 vs. Trip1 comparison when contrasted to the Dip0 vs. Trip0 comparison and the difference in size between the two groups.

Similar to the Dip1 vs. Trip1 comparison, we would expect Trip2 (2:3) to have a lower GH transgene concentration than Dip2 (2:2), which again was found to be the case in the present study (not significant in the current study, but a trend that was also observed in a previous study [44] using a different methodology). With the greater dose discrepancy (1/3 instead of 1/6), a surprising finding was that the Dip2 vs. Trip2 comparison identified only five DEGs, which is much fewer than the 274 found between Dip1 and Trip1. Possibly reflecting a biological maximum output of liver gene expression differences that the GH transgene can elicit. This is supported by the comparison between the Dip1 vs. Dip2 comparison with only 31 DEGs.

The two overlapping genes between the Dip1 vs. Trip1 and Dip2 vs. Trip2 comparisons, fish-egg lectin and type-4 ice-structuring protein, have immune and nutrition deprivation related functions. Fish-egg lectin has been linked with innate immune response in the rock bream [68], and has been shown to be up-regulated in the liver during pathogen challenges [69]. Type-4 ice-structuring protein shows abnormal regulation during nutritional deprivation in the liver [70–72]. In the present study, there appears to be a ploidy-by-transgene interaction with elevated expression of these gene in triploids with one or two transgene doses. The upregulation of these genes may be indicative of an adverse response to high levels of growth hormone in triploid coho salmon.

## Conclusions

Triploid coho salmon have hepatic gene expression profiles very similar to diploids, except for a few genes that play roles in nutritional deficiency and compensatory growth. Positive gene dosage effects, balanced by modification of cell size and number in triploids explains how polyploidy is so well tolerated in modern salmonids and possibly how tetraploidy was tolerated during the evolution of salmonids.

The growth hormone transgene was included in this study to evaluate the effects of a genomic and physiological (metabolic) modifier on triploids to investigate why coho salmon triploids tend to grow less rapidly compared to diploids. The growth hormone transgene significantly changed the expression profile of many hepatic genes, but did not respond in a linear dosage-dependent manner (potentially due to suppression of transgene expression in the Trip3 group). GH transgenesis played the largest role in global hepatic gene expression differences seen in this study, but the difference between having a single transgene versus multiple copies is much subtler. Overall, the growth hormone transgene consistently effected the expression of genes involved in metabolism regardless of ploidy.

The information provided in this study may be of value when evaluating the risks that triploid and transgenic salmon pose to wild populations, and suggests that new feeding protocols and tailored nutritional regimes would likely benefit the culture of polyploids and growth hormone transgenic fish.

## Methods

### Population and sample information

Seven groups of coho salmon were assayed for whole-liver gene expression in this study. Three of these groups were composed of diploid fish, while the other four were made up of triploid fish. The diploid and triploid fish were further sub-divided based on the number of copies of a GH transgenic locus they contained (from zero to two copies in the diploid individuals, and from zero to three copies in the triploid fish). The seven groups were given the following abbreviations: diploid without transgene: Dip0 (*n* = 6); diploid with a single copy of the transgene: Dip1 (*n* = 5); diploid with two copies of the transgene: Dip2 (n = 5); triploid without transgene: Trip0 (n = 6); triploid with a single copy of the transgene: Trip1 (*n* = 4); triploid with two copies of the transgene: Trip2 (n = 5); and triploid with three copies of the transgene: Trip3 (n = 4).

All of the coho salmon in this study were of Chehalis River, British Columbia strain background. The growth hormone transgene (strain M77) is inserted in a single position in the coho salmon genome and is described in [73, 74]. The different groups (above) were generated for another study and the methodology is described there [44]. Briefly, diploid coho salmon progeny were produced by crossing individuals possessing different transgene doses: 1) homozygotes for the growth hormone transgene were crossed with other homozygotes (producing progeny with two copies of the transgene), 2) homozygotes for the transgene (paternal source) with a wild-type to produce hemizygotes, or 3) two wild-types were crossed. Triploids were created from the same crosses (to reduce gene expression differences caused by allelic variation), but eggs were additionally pressure shocked to induce triploidy. Triploid progeny with two transgenes were generated by crossing homozygous transgenic females with wild-type males, and those with three transgenes were generated by crossing homozygous parents. Ploidy was determined by measurements of red blood cell diameter, as described in [42].

Fish husbandry and euthanasia followed guidelines from the Canadian Council for Animal Care under permit from Fisheries and Oceans Canada’s Pacific Regional Animal Care Committee (Ex.7.1, Pacific Region Animal Care Committee management procedure 3.7). The coho salmon progeny were raised in fresh, aerated well water (10 ± 0.5 °C) in 170 L tanks at a density of less than 5 kg/m^3^, with a simulated natural photoperiod. Feed (commercial Pacific salmon diets, Skretting Canada Ltd.) was provided in excess of satiation, initially at 6 times per day and decreasing to three times a day at the time of sampling. All fish were reared in contained facilities designed to prevent escape of transgenic animals to the wild.

### RNA extraction and sequencing

Each fish (201 days post first feeding) was euthanized in an anaesthetic bath containing 100 mg/L AquaLife TMS (MS-222) (Syndel Canada) buffered with 200 mg/L sodium bicarbonate (Sigma-Aldrich) prior to sampling. Tissues were rapidly team dissected (< 3 min per fish) and stored in RNAlater according to manufacturer’s instructions (Ambion). RNA was extracted using a Qiagen RNeasy kit following the manufacturer’s protocol. RNA samples were sent to the McGill University and Génome Québec Innovation Centre for Illumina TruSeq RNA library preparation and HiSeq 2500 PE125 sequencing on two lanes.

### Quality control and read alignment

Sequences were imported into CLC Genomics Workbench 9.5.4 [75] using the “paired-read” and “remove failed reads” options of the Illumina import feature of the software. Sequences were then aligned to the coho genome (NCBI accession: GCA_002021735.1), only mapping to the regions with genes using the same software and the RNA-seq analysis feature. Expression was calculated using the same feature, as fragments per kilobase of gene per million mapped reads (FPKM). Additional file 1: Table S6 describes the number of genes and other information related to the coho salmon genome, and Additional file 1: Table S7 describes the various parameters used for the alignment.

### Gene expression analysis

Three analyses were conducted to compare the gene expression between different groups. The first analysis was a pairwise comparison among all groups. The second, compared groups based on their ploidy (i.e. all diploid vs. all triploid). The third analysis was based on the copy number of transgenic growth hormone gene(s) in the genotypes of the different groups (e.g. none vs. one transgene copy, or one vs. two transgene copies, etc.). All analyses were performed using CLC Genomics Workbench 9.5.4.

First, the “unpaired, multi-group comparison” set up experiment feature in CLC Genomics Workbench was used to generate the groupings, followed by the “empirical analysis of digital gene expression (DGE)” feature to identify significant differentially expressed genes. In the “empirical analysis of DGE” settings, the total count filter cutoff was set to 5.0, which ignores genes (or features) with a cumulative read count across all samples of less than five when estimating the common dispersion component (such genes are expected to add noise to the estimate). The “estimate tagwise dispersions” setting was added to weight gene dispersion estimates based on the combination of gene-wise dispersions and the common dispersion component (an empirical Bayes procedure [76]). The “empirical analysis of DGE” feature is based on [77] and implemented in EdgeR [76]. The *p*-values generated from these analyses were corrected for multiple testing (i.e. FDR corrected) using methods described in [78]. Significance was defined as *p* < 0.055 after correction. A principal component analysis of the gene expression data was also performed. All analyses were conducted using original expression values. Correlation coefficients were calculated in a pair-wise fashion between individuals using expression values in LibreOffice Calc [79].

### Gene ontology

The Blast2GO suite (version 5 Basic) [80] was used to assign gene ontologies to differentially expressed genes. First coho salmon protein sequences (associated with the genome, see above) were downloaded from the NCBI and aligned to the UniProtKB (Swiss-Prot) database (downloaded on March 28, 2018) [81] using BLASTP-fast [82] within Blast2GO (maximum hits 5, with all other parameters default). The mapping and annotation steps followed using the default parameters. The annotation file (.annot) was then exported and isoforms were removed to eliminate bias toward proteins without isoforms using a custom python script (Additional file 10).

The differentially expressed genes were then tested for GO term enrichment using a Fisher’s exact test in Blast2GO (default settings). As the GO database was generated using proteins and the gene expression data was reported as genes, it was necessary to convert the gene names to the corresponding protein names. This was performed with a custom script (Additional file 10) applied to the gene annotation file (gff) downloaded from the NCBI. Isoforms were not included in this data as they may cause artificial enrichment of categories. Information on GO terms was obtained from QuickGO [83].

There were several hundred enriched GO terms for some of the comparisons, and thus three approaches were taken to reduce the complexity of the data. The first approach produced an enriched graph in Blast2GO and retrieved the categories from the second and third levels of the biological function categories. The second approach was to reduce the complexity using the REVIGO software [84] with an allowed similarity of 0.5, (*Danio rerio* database). The output from this analysis was further subset to report only categories that had dispensability scores of less than or equal to 0.1. This score reports the most different GO categories that overlap the least. Both of these methods have biases in reporting, but are useful in assessing major categories that are differentially expressed. The third method to reduce the complexity of enriched GO terms was to generate a tree map in REVIGO to summarize the important differences between the groups. GO categories were reported for each gene using custom python scripts (Additional file 10). Only the biological process GO categories are reported, but the other categories can be found in the Additional file 2.

## Additional files

Additional file 1: Supporting Tables. This is a document file with supporting/supplementary tables. (ZIP 13 kb)

Additional file 2: Supporting Information. This is a spreadsheet file with information on RNA-seq alignments, significant differentially expressed genes for the various comparisons, growth hormone (GH and GH2) gene expression, principal component analyses results, enriched GO categories, the REVIGO reduced GO categories, and summary information. (ZIP 4703 kb)

Additional file 3: **Figure S1.** Enriched Gene Ontology Categories Between Dip0 and Dip1. After an analysis of GO term enrichment (Fisher’s exact test) was performed on the DEGs between Dip0 and Dip1, enriched GO term numbers (433 enriched categories) were reduced using the software REVIGO. The size of each rectangle is based on the false discovery rate corrected *p*-values. The REVIGO software uses an algorithm similar to hierarchical clustering based on semantic similarity between GO terms (i.e. proximity of terms in the GO hierarchy). Each displayed GO term is a representative from a cluster of similar GO terms (i.e. semantically similar terms) and is joined into superclusters with representatives having the same colour. (TIF 1660 kb)

Additional file 4: **Figure S2.** Enriched Gene Ontology Categories Between Trip0 and Trip1. After an analysis of GO term enrichment (Fisher’s exact test) was performed on the DEGs between Trip0 and Trip1, enriched GO term numbers (567 enriched categories) were reduced using the software REVIGO. The size of each rectangle is based on the false discovery rate corrected *p*-values. The REVIGO software uses an algorithm similar to hierarchical clustering based on semantic similarity between GO terms (i.e. proximity of terms in the GO hierarchy). Each displayed GO term is a representative from a cluster of similar GO terms (i.e. semantically similar terms) and is joined into superclusters with representatives having the same colour. (TIF 1612 kb)

Additional file 5: **Figure S3.** Enriched Gene Ontology Categories Between Dip0 and Dip1. After GO term enrichment (Fisher’s exact test) was performed on the DEGs between Dip0 and Dip1, enriched GO term complexity (433 enriched categories) was reduced using the software REVIGO. Each displayed GO term is a representative from a cluster of similar GO terms and is joined into superclusters with representatives having the same colour. The resulting tree map category sizes are based on the number of DEGs in each GO category. This figure differs from Additional file 3: Figure S1 in terms of the types of categories shown and the relative size of categories because the number of DEGs in a category did not necessarily correlate with the *p*-value. For example, a category with only five representative genes, can be more significantly enriched with four DEGs, than a category with thousands of representative genes with hundreds of DEGs in the category. (TIF 1423 kb)

Additional file 6: **Figure S4.** Enriched Gene Ontology Categories Between Trip0 and Trip1. After GO term enrichment (Fisher’s exact test) was performed on the DEGs between Trip0 and Trip1, enriched GO term complexity (567 enriched categories) was reduced using the software REVIGO. Each displayed GO term is a representative from a cluster of similar GO terms and is joined into superclusters with representatives having the same colour. The resulting tree map category sizes are based on the number of DEGs in each GO category. This figure differs from Additional file 4: Figure S2 in terms of the types of categories shown and the relative size of categories because the number of DEGs in a category did not necessarily correlate with the p-value. For example, a category with only five representative genes, can be more significantly enriched with four DEGs, than a category with thousands of representative genes with hundreds of DEGs in the category. (TIF 1430 kb)

Additional file 7: **Figure S5.** Enriched Gene Ontology Categories Between Dip1 and Trip1. After GO term enrichment (Fisher’s exact test) was performed on the DEGs between Dip1 and Trip1, enriched GO term complexity (182 enriched categories) was reduced using the software REVIGO. Each displayed GO term is a representative from a cluster of similar GO terms and is joined into superclusters with representatives having the same colour. The size of each rectangle is based on the false discovery rate corrected *p*-values. (TIF 914 kb)

Additional file 8: **Figure S6.** Enriched Gene Ontology Categories Between Dip1 and Trip1. After GO term enrichment (Fisher’s exact test) was performed on the DEGs between Dip1 and Trip1, enriched GO term complexity (182 enriched categories) was reduced using the software REVIGO. Each displayed GO term is a representative from a cluster of similar GO terms and is joined into superclusters with representatives having the same colour. The resulting tree map category sizes are based on the number of DEGs in each GO category. This figure differs from Additional file 7: Figure S5 in terms of the types of categories shown and the relative size of categories because the number of DEGs in a category did not necessarily correlate with the p-value. For example, a category with only five representative genes, can be more significantly enriched with four DEGs, than a category with thousands of representative genes with hundreds of DEGs in the category. (TIF 977 kb)

Additional file 9: **Figure S7.** Transgenic Growth Hormone Gene Expression and Growth from Previous Study. A) Figure reproduced with permission from [46]. The number of transgenes is shown on the x-axis and the average weight of each group is shown on the y-axis (201 days post first feeding). The 2n group corresponds to Dip in the current study (non-transgenic, *n* = 40; 1 transgene dose, *n* = 67; 2 transgenes doses, *n* = 17). The 3n group corresponds to Trip in the current study (non-transgenic, *n* = 25; 1 transgene dose, *n* = 19; 2 transgene doses, *n* = 27; and 3 transgenes doses, *n* = 26). The different letters represent significant differences among groups and transgene dosages. Values are mean ± SE. B) mRNA levels of growth hormone in the liver of coho salmon, which was adapted with permission from [46]. On the x-axis are the different groups (2 N - diploid, 3 N - triploid) with increasing numbers of the growth hormone transgene doses (0 = non-transgenic to 3 = three copies), *n* = 8–11. On the y-axis, the level of mRNA is measured as the mean of each group ± SE value determined by Q-PCR. The different letters represent groups that are significantly different. (TIF 161 kb)

Additional file 10: Python Scripts. This is a compressed folder with all of the scripts used in the analyses in this study. It contains a readme file to describe the use of these scripts. (ZIP 23 kb)

## Abbreviations

1R: First vertebrate genome duplication
2R: Second vertebrate genome duplication
3R: Teleost-specific genome duplication
4R: Salmonid-specific genome duplication
DEG: Differentially expressed gene
Dip0: Diploid coho salmon without the growth hormone transgene
Dip1: Diploid coho salmon with a single growth hormone transgene
Dip2: Diploid coho salmon with two growth hormone transgenes
FPKM: Fragments per kilobase of transcript per million mapped reads
GH: Growth hormone
GO: Gene ontology
Trip0: Triploid coho salmon without the growth hormone transgene
Trip1: Triploid coho salmon with a single growth hormone transgene
Trip2: Triploid coho salmon with two growth hormone transgenes
Trip3: Triploid coho salmon with three growth hormone transgenes
