# Supplementary material for: Effect of triploidy on liver gene expression in coho salmon (Oncorhynchus kisutch) under different metabolic states
Source: BMC Genomics. 2019 May 3;20:336. doi: 10.1186/s12864-019-5655-8 (PMC6500012; doi:10.1186/s12864-019-5655-8)
Supplement: Supplementary file 1 — Supporting Tables. This is a document file with supporting/supplementary tables. (ZIP 13 kb) [file 12864_2019_5655_MOESM1_ESM.zip › S3 File/SupplementalFile3.docx]

Table S1. Read and Alignment Statistics

|  | **Before Alignments** | **After Alignments (Paired + Unpaired)** | **Average Aligned**  **(Paired + Unpaired)** |
| --- | --- | --- | --- |
| **Total number of reads in experiment** | 1078848730 | 818544058 + 100789746 | 85.21% |
| **Average number of reads per individual** | 30824249 | 23386973 + 2879707 | - |
| **Standard deviation** | 4632113 | 3452324 + 1146307 | - |

Table S2. Differentially Expressed Genes Between Dip0 and Trip0

| **NCBI ID** | **Common Name** | **Dip0 FPKM** | **Trip0 FPKM** | **P-value** |
| --- | --- | --- | --- | --- |
| 109905597 | selenium binding protein 1 | 95.82 | 45.3 | 0.0000374 |
| 109896853 | eukaryotic initiation factor 4A-I | 16.84 | 37.19 | 0.000135 |
| 109893262 | eukaryotic translation initiation factor 1 | 41.29 | 80.86 | 0.00123 |
| 109906549 | perilipin-2-like | 3.9 | 13.09 | 0.00134 |
| 109882155 | NOP58 ribonucleoprotein | 6.51 | 16.69 | 0.00696 |
| 109905743 | heat shock cognate 70 kDa protein-like | 40.78 | 101.27 | 0.02 |
| 109879509 | transmembrane 4 L six family member 4 | 91.65 | 57.76 | 0.02 |
| 109893008 | early growth response protein 1-like | 1.38 | 6.22 | 0.04 |
| 109871614 | ATP-binding cassette sub-family F member 2-like | 2.71 | 9.03 | 0.04 |
| 109899801 | acyl-CoA desaturase-like | 9.63 | 25.69 | 0.04 |
| 109879273 | gamma-aminobutyric acid receptor-associated protein-like 1 | 24.68 | 11.57 | 0.04 |
| 109895401 | glycine cleavage system H protein, mitochondrial-like | 45.92 | 24.51 | 0.04 |
| 4955089 | ATP synthase F0 subunit 8 | 1499.02 | 912.74 | 0.04 |
| 109872894 | nucleophosmin-like | 14.12 | 25.72 | 0.05 |
| 109900308 | thyroxine 5-deiodinase-like | 24.9 | 11.31 | 0.05 |
| 109868384 | phosphatidylserine decarboxylase proenzyme, mitochondrial-like | 32.87 | 93.84 | 0.05 |

Table S3. Graph Based Analysis of GO Categories of DEG Between Several Comparisons

|  |  |  | **Dip0vsDip1** | | **Trip0vsTrip1** | | **Dip1vsTrip1** | |
| --- | --- | --- | --- | --- | --- | --- | --- | --- |
| **Level** | **GO ID** | **GO Name** | Dip0 (+) | Dip1 (+) | Trip0 (+) | Trip1 (+) | Dip1 (+) | Trip1 (+) |
| 2 | GO:0008152 | metabolic process | 145 | **377** | 263 | **362** | **215** | 18 |
| 3 | GO:0071704 | organic substance metabolic process | 138 | **358** | 256 | **340** | **212** | 16 |
| 3 | GO:0044237 | cellular metabolic process | 134 | **353** | 245 | **336** | **209** | 16 |
| 3 | GO:0044238 | primary metabolic process | 128 | **343** | 249 | **321** | **212** | 15 |
| 3 | GO:0006807 | nitrogen compound metabolic process | 117 | **317** | 229 | **298** | **206** | 14 |
| 3 | GO:0009058 | biosynthetic process | 90 | **212** | 155 | **213** | **185** | 12 |
| 3 | GO:0009056 | catabolic process | 51 | **103** | 75 | **116** | **60** | 5 |
| 3 | GO:0044281 | small molecule metabolic process | 54 | **190** | 64 | **218** | 0 | 0 |
| 3 | GO:0042221 | response to chemical | 89 | **177** | 134 | **175** | 0 | 0 |
| 3 | GO:0006950 | response to stress | 73 | **142** | 124 | **126** | 0 | 0 |
| 3 | GO:0055114 | oxidation-reduction process | 35 | **124** | 35 | **123** | 0 | 0 |
| 3 | GO:0042445 | hormone metabolic process | 8 | **14** | 10 | **14** | 0 | 0 |
| 2 | GO:0098754 | detoxification | 5 | **13** | 4 | **16** | 0 | 0 |
| 3 | GO:1990748 | cellular detoxification | 4 | **13** | 4 | **15** | 0 | 0 |
| 3 | GO:0072376 | protein activation cascade | **8** | 5 | **18** | 2 | 0 | 0 |
| 2 | GO:0001906 | cell killing | 6 | **10** | **10** | 6 | 0 | 0 |
| 3 | GO:0042440 | pigment metabolic process | 5 | **8** | 5 | **8** | 0 | 0 |
| 3 | GO:1904582 | positive regulation of intracellular mRNA localization | **3** | 1 | **2** | 1 | 0 | 0 |
| 3 | GO:0044085 | cellular component biogenesis | 0 | 0 | 0 | 0 | **93** | 3 |
| 3 | GO:0009892 | negative regulation of metabolic process | 0 | 0 | 0 | 0 | **78** | 2 |
| 3 | GO:0002262 | myeloid cell homeostasis | 0 | 0 | 0 | 0 | **12** | 1 |
| 3 | GO:0051918 | negative regulation of fibrinolysis | 0 | 0 | **5** | 0 | **3** | 0 |
| 3 | GO:0007568 | aging | 0 | 0 | 14 | **26** | 0 | 0 |
| 3 | GO:0019748 | secondary metabolic process | 0 | 0 | 4 | **7** | 0 | 0 |
| 3 | GO:0006457 | protein folding | 0 | 0 | 10 | **11** | 0 | 0 |
| 3 | GO:1900046 | regulation of hemostasis | 0 | 0 | **16** | 1 | 0 | 0 |
| 3 | GO:1900048 | positive regulation of hemostasis | 0 | 0 | **8** | 1 | 0 | 0 |
| 3 | GO:1900047 | negative regulation of hemostasis | 0 | 0 | **13** | 0 | 0 | 0 |
| 3 | GO:0001909 | leukocyte mediated cytotoxicity | 0 | 0 | **9** | 4 | 0 | 0 |
| 3 | GO:1904874 | positive regulation of telomerase RNA localization to Cajal body | 0 | 0 | **3** | 0 | 0 | 0 |
| 3 | GO:0035732 | nitric oxide storage | 0 | **2** | 0 | 0 | 0 | 0 |
| 3 | GO:0031640 | killing of cells of other organism | 3 | **4** | 0 | 0 | 0 | 0 |

The “Level” column is based on the position of the GO category in the enriched graph produced by Blast2GO. The last three columns (e.g. Dip0vsDip1) display the number of genes upregulated in the different groups (e.g. Dip0 or Dip1) relative to the other group in the comparison. The group with the most upregulated genes in a comparison is shown in bold text.

Table S4. REVIGO Based Analysis of GO Categories of DEG Between Several Comparisons (Based on Dispensability Score <= 0.1)

|  |  |  | **Dip0vsDip1** | | **Trip0vsTrip1** | | **Dip1vsTrip1** | |
| --- | --- | --- | --- | --- | --- | --- | --- | --- |
| **DS** | **GO ID** | **GO Name** | Dip0 (+) | Dip1 (+) | Trip0 (+) | Trip1 (+) | Dip1 (+) | Trip1 (+) |
| 0 | GO:0008152 | metabolic process | 145 | **377** | 263 | **362** | **215** | 18 |
| 0 | GO:0097421 | liver regeneration | 3 | **5** | **7** | 3 | **13** | 0 |
| 0 | GO:0006412 | translation | 12 | **48** | **39** | 13 | **162** | 0 |
| 0 | GO:0042886 | amide transport | 0 | 0 | 0 | 0 | **56** | 3 |
| 0 | GO:0034101 | erythrocyte homeostasis | 0 | 0 | 0 | 0 | **12** | 0 |
| 0 | GO:1904400 | response to Thyroid stimulating hormone | 0 | 0 | 0 | 0 | **3** | 0 |
| 0 | GO:0042254 | ribosome biogenesis | 0 | 0 | **36** | 1 | **77** | 0 |
| 0 | GO:0055114 | oxidation-reduction process | 35 | **124** | 35 | **123** | 0 | 0 |
| 0.09 | GO:0006091 | generation of precursor metabolites and energy | 13 | **89** | 19 | **72** | 0 | 0 |
| 0.09 | GO:0051186 | cofactor metabolic process | 31 | **61** | 24 | **88** | 0 | 0 |
| 0.09 | GO:1901615 | organic hydroxy compound metabolic process | 19 | **30** | 25 | **38** | 0 | 0 |
| 0.09 | GO:0072593 | reactive oxygen species metabolic process | 11 | **21** | 16 | **23** | 0 | 0 |
| 0.1 | GO:0016999 | antibiotic metabolic process | 5 | **18** | 5 | **28** | 0 | 0 |
| 0.03 | GO:0042407 | cristae formation | 0 | **22** | 0 | **20** | **5** | 0 |
| 0.04 | GO:0007007 | inner mitochondrial membrane organization | 0 | **25** | 0 | **20** | **5** | 0 |
| 0.04 | GO:1902600 | hydrogen ion transmembrane transport | 2 | **42** | 3 | **32** | **8** | 0 |
| 0.07 | GO:0006805 | xenobiotic metabolic process | 7 | **14** | 5 | **14** | 0 | 0 |
| 0.07 | GO:0042133 | neurotransmitter metabolic process | 9 | **13** | 10 | **20** | 0 | 0 |
| 0.04 | GO:0015671 | oxygen transport | 0 | **18** | 0 | **17** | 0 | 0 |
| 0.07 | GO:0006801 | superoxide metabolic process | 5 | **11** | 4 | **9** | 0 | 0 |
| 0.08 | GO:0072376 | protein activation cascade | **8** | 5 | **18** | 2 | 0 | 0 |
| 0.09 | GO:0035383 | thioester metabolic process | 6 | **9** | 2 | **19** | 0 | 0 |
| 0 | GO:0001906 | cell killing | 6 | **10** | **10** | 6 | 0 | 0 |
| 0.1 | GO:0045454 | cell redox homeostasis | 0 | **14** | 1 | **10** | 0 | 0 |
| 0.06 | GO:0042744 | hydrogen peroxide catabolic process | 0 | **5** | 0 | **7** | 0 | 0 |
| 0.03 | GO:0043589 | skin morphogenesis | 2 | **3** | 2 | **4** | 0 | 0 |

The “DS” column displays dispensability scores calculated in the REVIGO software. The last three columns (e.g. Dip0vsDip1) display the number of genes upregulated in the different groups (e.g. Dip0 or Dip1) relative to the other group in the comparison. The group with the most upregulated genes in a comparison is shown in bold text.

Table S5. Top 20 Differentially Expressed Genes Between Dip1 and Trip1 (not overlapping with Dip0 and Dip1 or Trip0 and Trip1)

| **NCBI ID** | **Common Name** | **Dip1 FPKM** | **Trip1 FPKM** | **P-value** |
| --- | --- | --- | --- | --- |
| 109865995 | glucose-6-phosphatase-like | 56.75 | 18.14 | 0.0002 |
| 109898698 | 60S ribosomal protein L3 | 438.66 | 290.86 | 0.0003 |
| 109883581 | 60S ribosomal protein L23-like | 678.82 | 380.2 | 0.0003 |
| 109900064 | fatty acid synthase-like | 2.86 | 29.54 | 0.0003 |
| 109906755 | 40S ribosomal protein S19 | 474.12 | 326.16 | 0.0004 |
| 109886302 | 40S ribosomal protein S9 | 497.92 | 296.68 | 0.0006 |
| 109876330 | 40S ribosomal protein S8-like | 521.77 | 359.17 | 0.0011 |
| 109907378 | 60S ribosomal protein L15 | 573.61 | 391.12 | 0.0011 |
| 109894019 | ribosomal protein S3A | 674.96 | 428.29 | 0.0013 |
| 109895399 | 60S ribosomal protein L13 | 552.86 | 330.97 | 0.0015 |
| 109903681 | elongation factor 1-alpha, oocyte form-like | 2055.2 | 1386.14 | 0.0015 |
| 109867828 | 60S ribosomal protein L27a | 1007.77 | 663.88 | 0.0016 |
| 109894672 | 60S ribosomal protein L13a | 1042.18 | 692.75 | 0.0019 |
| 109903490 | 60S ribosomal protein L15-like | 599.32 | 368.85 | 0.0019 |
| 109906632 | 60S ribosomal protein L34 | 447.48 | 290.58 | 0.0019 |
| 109903732 | 40S ribosomal protein S5 | 458.99 | 295.3 | 0.0019 |
| 109899796 | 40S ribosomal protein S24 | 445.51 | 303.75 | 0.0019 |
| 109880473 | 40S ribosomal protein S8 | 833.25 | 562.07 | 0.0019 |
| 109870699 | 60S ribosomal protein L31 | 252.4 | 168.07 | 0.0019 |
| 109883896 | ribosomal protein L7 | 1098.19 | 724.27 | 0.0019 |

Table S6. Reference Genome Information

| **Number of Nucleotides** | 2369915580 |
| --- | --- |
| **Number of Genes** | 46109 |
| **Number of Transcripts** | 57579 |

Table S7. Alignment and Gene Expression Parameters

| **Alignment Parameters** |  |
| --- | --- |
| Mismatch cost | 2 |
| Insertion cost | 3 |
| Deletion cost | 3 |
| Minimum length fraction | 0.9 |
| Minimum similarity fraction | 0.9 |
| Maximum number of hits for a read | 1 |
| **Gene Expression Parameters** |  |
| Count paired reads as two | No |
| Expression value | RPKM (FPKM) |
| Use EM estimation | Yes |
