# Supplementary figures and images for: Effect of triploidy on liver gene expression in coho salmon (Oncorhynchus kisutch) under different metabolic states
Source: BMC Genomics. 2019 May 3;20:336. doi: 10.1186/s12864-019-5655-8 (PMC6500012; doi:10.1186/s12864-019-5655-8)

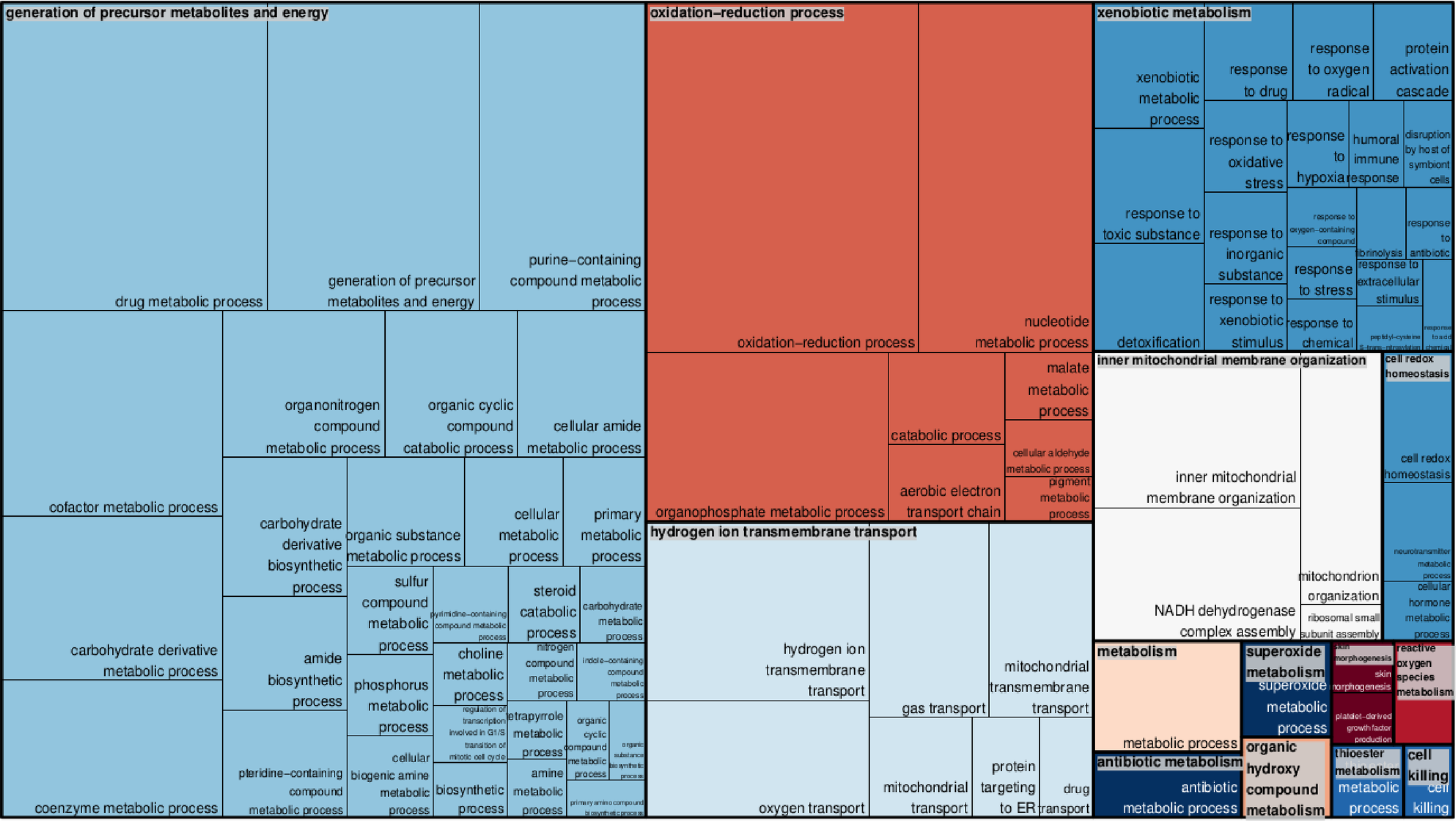

Supplement: Supplementary file 3 — Figure S1. Enriched Gene Ontology Categories Between Dip0 and Dip1. After an analysis of GO term enrichment (Fisher’s exact test) was performed on the DEGs between Dip0 and Dip1, enriched GO term numbers (433 enriched categories) were reduced using the software REVIGO. The size of each rectangle is based on the false discovery rate corrected p-values. The REVIGO software uses an algorithm similar to hierarchical clustering based on semantic similarity between GO terms (i.e. proximity of terms in the GO hierarchy). Each displayed GO term is a representative from a cluster of similar GO terms (i.e. semantically similar terms) and is joined into superclusters with representatives having the same colour. (TIF 1660 kb) [file 12864_2019_5655_MOESM3_ESM.tif]

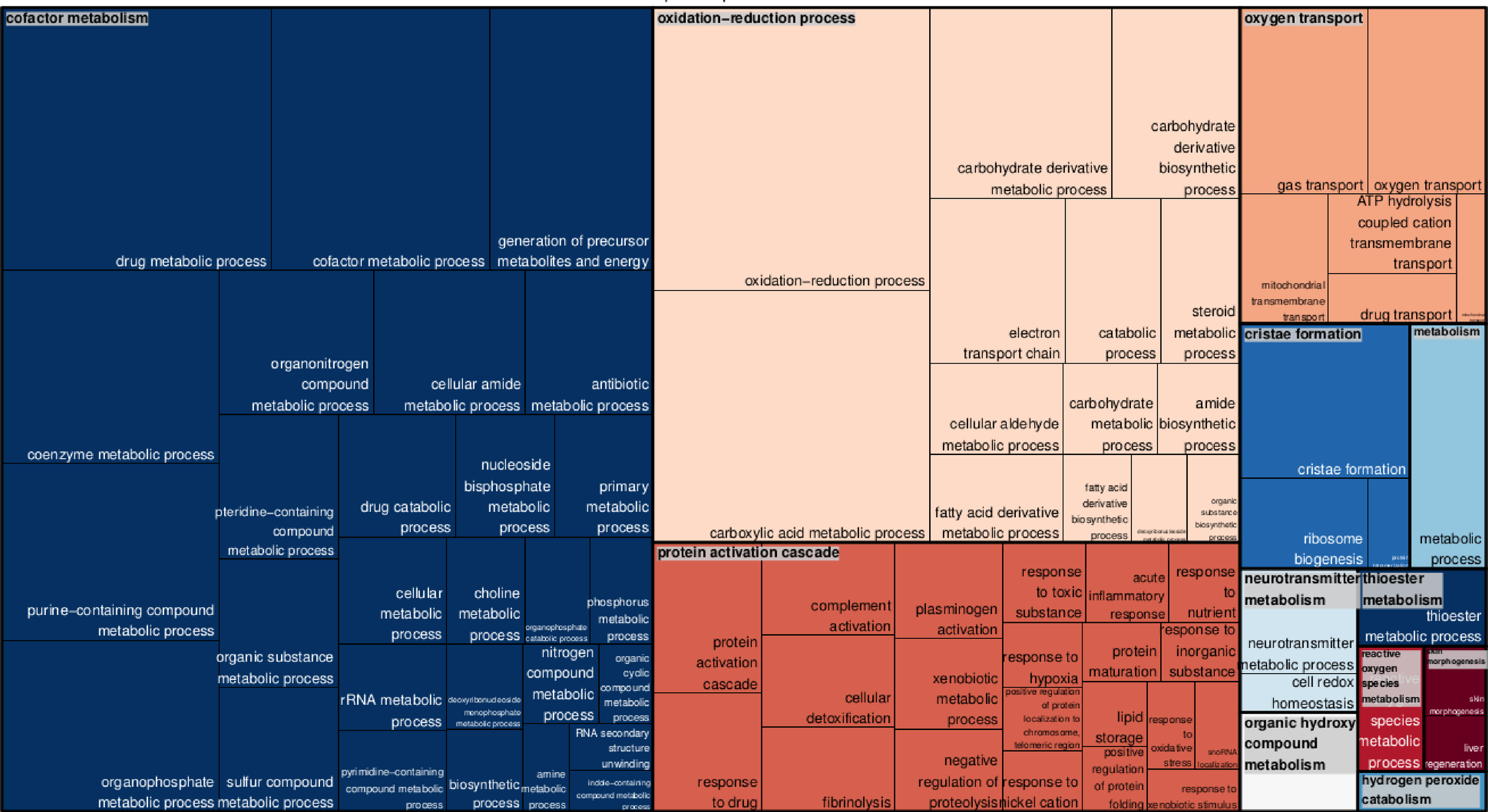

Supplement: Supplementary file 4 — Figure S2. Enriched Gene Ontology Categories Between Trip0 and Trip1. After an analysis of GO term enrichment (Fisher’s exact test) was performed on the DEGs between Trip0 and Trip1, enriched GO term numbers (567 enriched categories) were reduced using the software REVIGO. The size of each rectangle is based on the false discovery rate corrected p-values. The REVIGO software uses an algorithm similar to hierarchical clustering based on semantic similarity between GO terms (i.e. proximity of terms in the GO hierarchy). Each displayed GO term is a representative from a cluster of similar GO terms (i.e. semantically similar terms) and is joined into superclusters with representatives having the same colour. (TIF 1612 kb) [file 12864_2019_5655_MOESM4_ESM.tif]

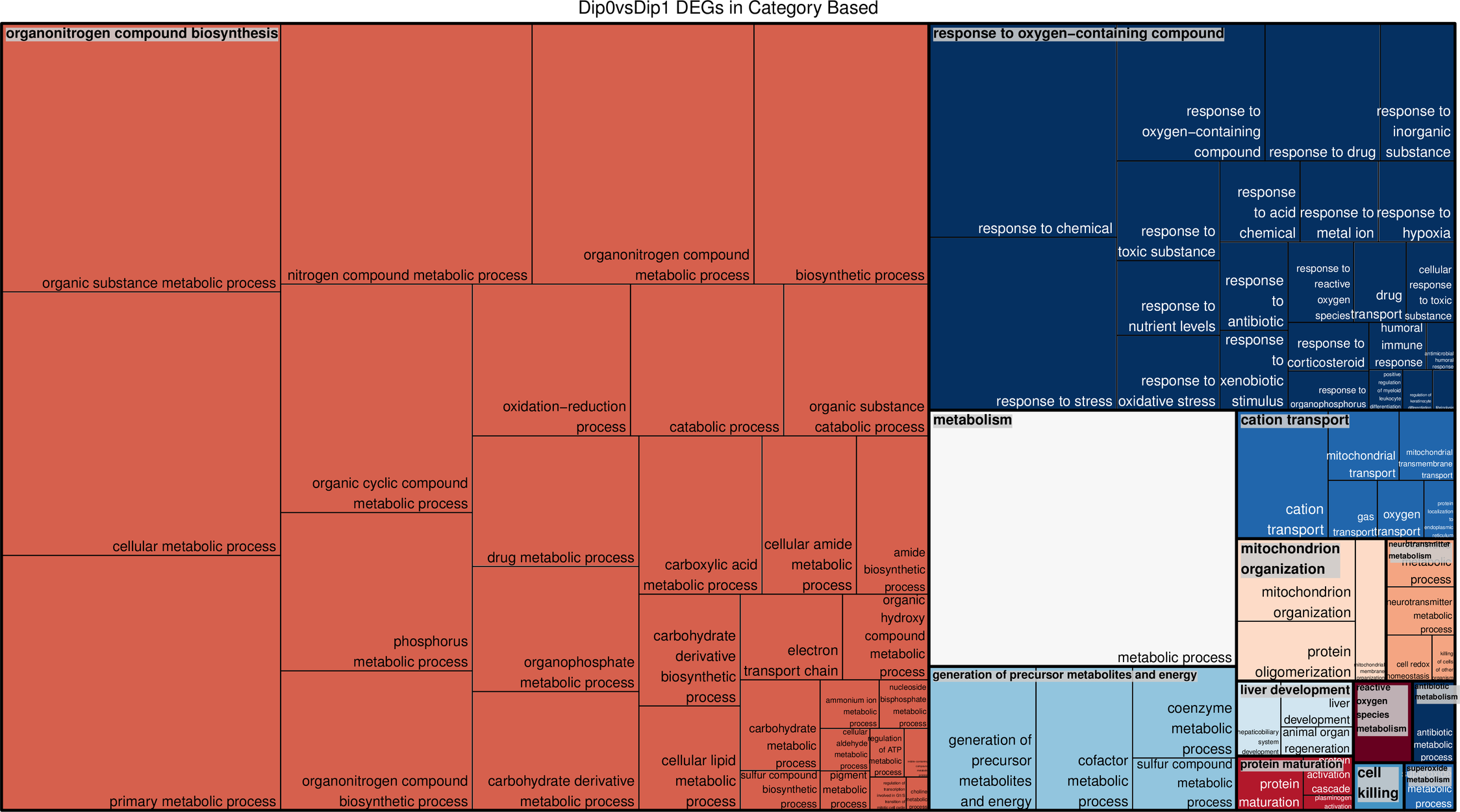

Supplement: Supplementary file 5 — Figure S3. Enriched Gene Ontology Categories Between Dip0 and Dip1. After GO term enrichment (Fisher’s exact test) was performed on the DEGs between Dip0 and Dip1, enriched GO term complexity (433 enriched categories) was reduced using the software REVIGO. Each displayed GO term is a representative from a cluster of similar GO terms and is joined into superclusters with representatives having the same colour. The resulting tree map category sizes are based on the number of DEGs in each GO category. This figure differs from Additional file 3: Figure S1 in terms of the types of categories shown and the relative size of categories because the number of DEGs in a category did not necessarily correlate with the p-value. For example, a category with only five representative genes, can be more significantly enriched with four DEGs, than a category with thousands of representative genes with hundreds of DEGs in the category. (TIF 1423 kb) [file 12864_2019_5655_MOESM5_ESM.tif]

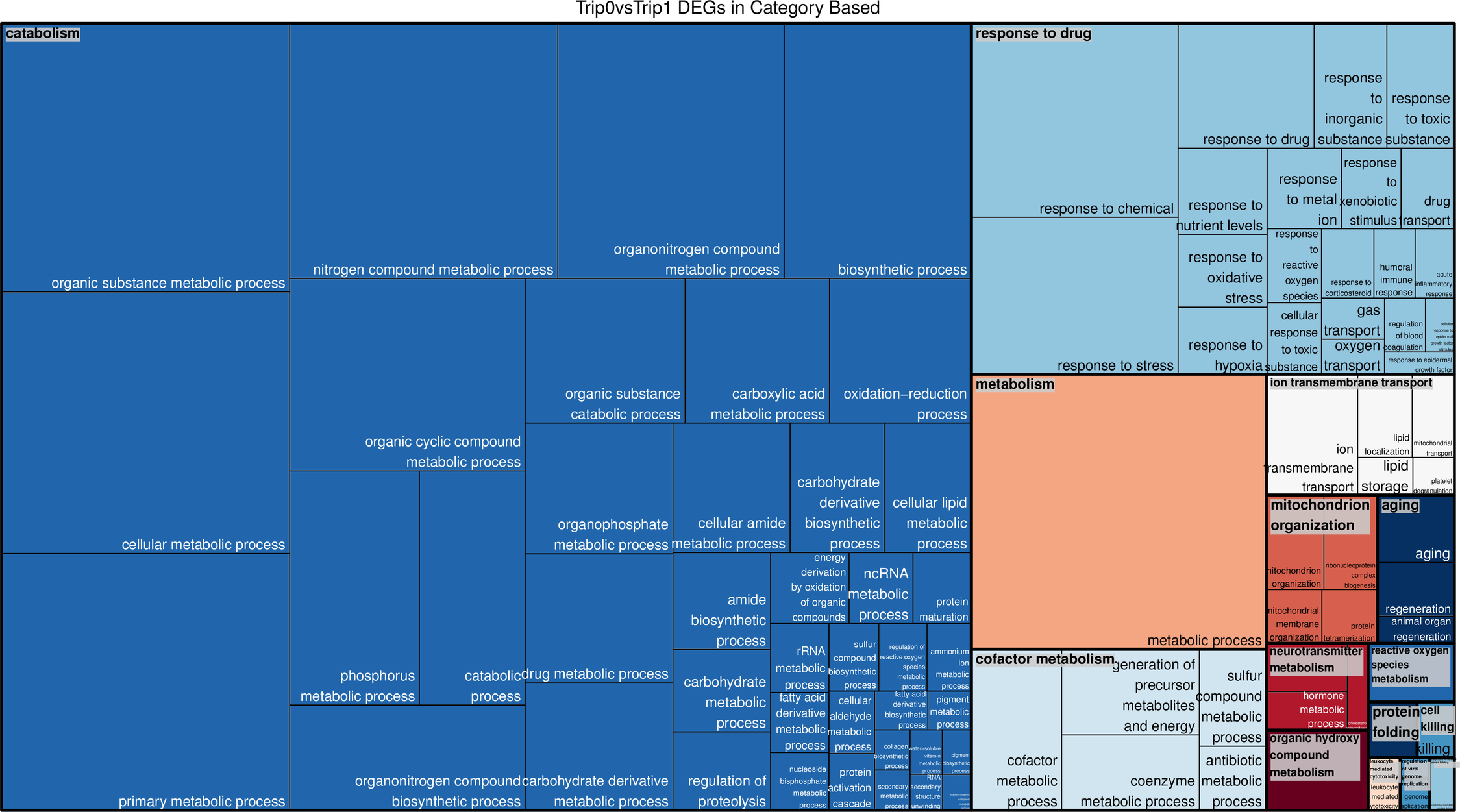

Supplement: Supplementary file 6 — Figure S4. Enriched Gene Ontology Categories Between Trip0 and Trip1. After GO term enrichment (Fisher’s exact test) was performed on the DEGs between Trip0 and Trip1, enriched GO term complexity (567 enriched categories) was reduced using the software REVIGO. Each displayed GO term is a representative from a cluster of similar GO terms and is joined into superclusters with representatives having the same colour. The resulting tree map category sizes are based on the number of DEGs in each GO category. This figure differs from Additional file 4: Figure S2 in terms of the types of categories shown and the relative size of categories because the number of DEGs in a category did not necessarily correlate with the p-value. For example, a category with only five representative genes, can be more significantly enriched with four DEGs, than a category with thousands of representative genes with hundreds of DEGs in the category. (TIF 1430 kb) [file 12864_2019_5655_MOESM6_ESM.tif]

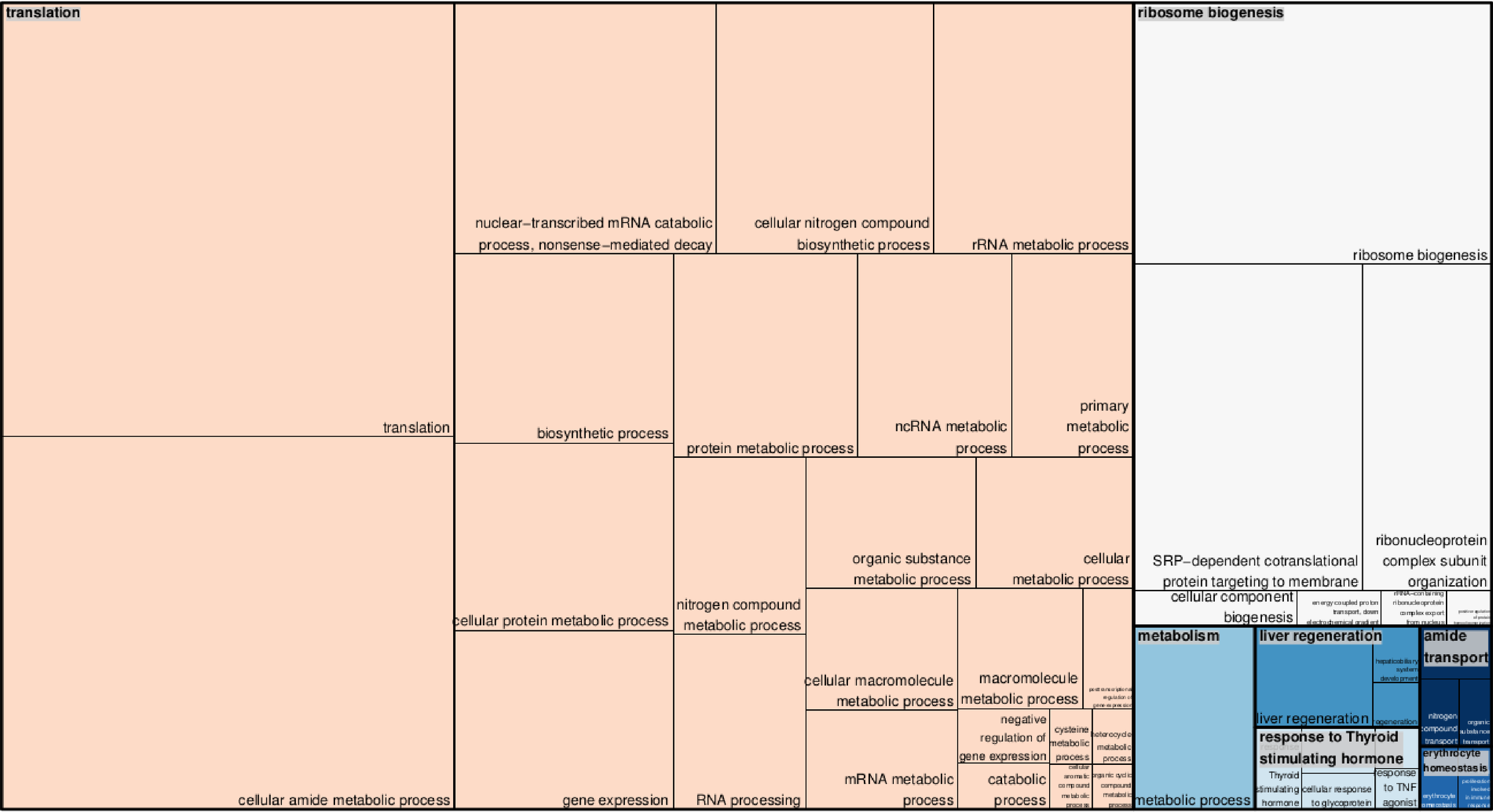

Supplement: Supplementary file 7 — Figure S5. Enriched Gene Ontology Categories Between Dip1 and Trip1. After GO term enrichment (Fisher’s exact test) was performed on the DEGs between Dip1 and Trip1, enriched GO term complexity (182 enriched categories) was reduced using the software REVIGO. Each displayed GO term is a representative from a cluster of similar GO terms and is joined into superclusters with representatives having the same colour. The size of each rectangle is based on the false discovery rate corrected p-values. (TIF 914 kb) [file 12864_2019_5655_MOESM7_ESM.tif]

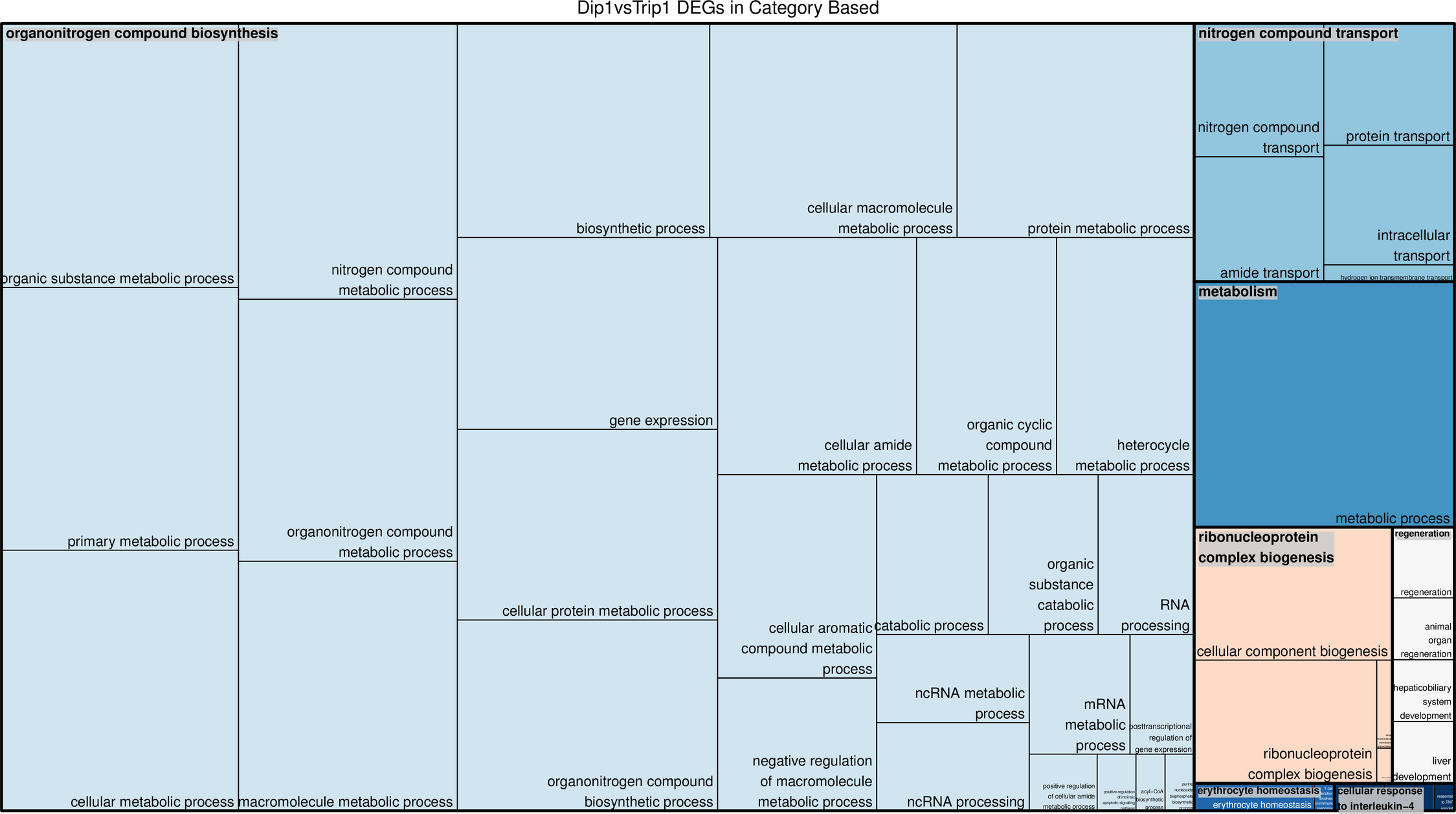

Supplement: Supplementary file 8 — Figure S6. Enriched Gene Ontology Categories Between Dip1 and Trip1. After GO term enrichment (Fisher’s exact test) was performed on the DEGs between Dip1 and Trip1, enriched GO term complexity (182 enriched categories) was reduced using the software REVIGO. Each displayed GO term is a representative from a cluster of similar GO terms and is joined into superclusters with representatives having the same colour. The resulting tree map category sizes are based on the number of DEGs in each GO category. This figure differs from Additional file 7: Figure S5 in terms of the types of categories shown and the relative size of categories because the number of DEGs in a category did not necessarily correlate with the p-value. For example, a category with only five representative genes, can be more significantly enriched with four DEGs, than a category with thousands of representative genes with hundreds of DEGs in the category. (TIF 977 kb) [file 12864_2019_5655_MOESM8_ESM.tif]

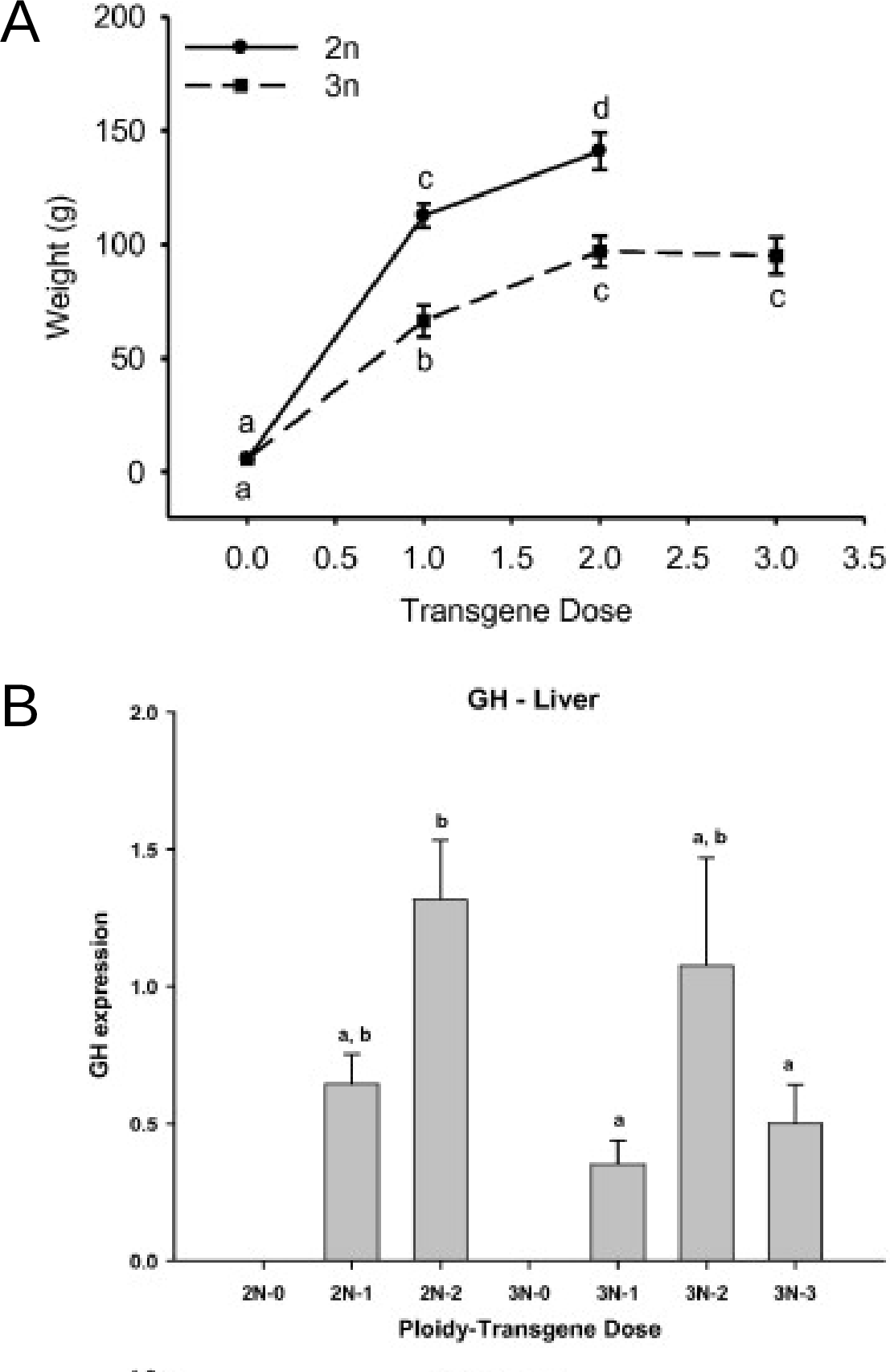

Supplement: Supplementary file 9 — Figure S7. Transgenic Growth Hormone Gene Expression and Growth from Previous Study. A) Figure reproduced with permission from [46]. The number of transgenes is shown on the x-axis and the average weight of each group is shown on the y-axis (201 days post first feeding). The 2n group corresponds to Dip in the current study (non-transgenic, n = 40; 1 transgene dose, n = 67; 2 transgenes doses, n = 17). The 3n group corresponds to Trip in the current study (non-transgenic, n = 25; 1 transgene dose, n = 19; 2 transgene doses, n = 27; and 3 transgenes doses, n = 26). The different letters represent significant differences among groups and transgene dosages. Values are mean ± SE. B) mRNA levels of growth hormone in the liver of coho salmon, which was adapted with permission from [46]. On the x-axis are the different groups (2 N - diploid, 3 N - triploid) with increasing numbers of the growth hormone transgene doses (0 = non-transgenic to 3 = three copies), n = 8–11. On the y-axis, the level of mRNA is measured as the mean of each group ± SE value determined by Q-PCR. The different letters represent groups that are significantly different. (TIF 161 kb) [file 12864_2019_5655_MOESM9_ESM.tif]
